# Critical limitations compromise the conclusions of a recent meta-analysis regarding spinal manipulation and migraine: a commentary

**DOI:** 10.1186/s13643-025-02849-5

**Published:** 2025-10-23

**Authors:** Robert J. Trager, Marc A. Bronson, Clinton J. Daniels, Stephen M. Perle

**Affiliations:** 1 Connor Whole Health, University Hospitals Cleveland Medical Center, Cleveland, OH USA; 2 Department of Family Medicine and Community Health, Case Western Reserve University School of Medicine, Cleveland, OH USA; 3 Department of Biostatistics and Bioinformatics Clinical Research Training Program, Duke University School of Medicine, Durham, NC USA; 4 Private Practice, Kirkland Lake, ON Canada; 5 Rehabilitation Care Services, VA Puget Sound Health Care System, Tacoma, WA USA; 6 Rehabilitation Medicine, University of Washington, Seattle, WA USA; 7 Big Data Interrogation Group, Health Sciences University, Bournemouth, UK; 8 School of Allied Health, Murdoch University, Murdoch, WA Australia

**Keywords:** Chiropractic, Spinal manipulation, Migraine, Headache disorders, Systematic review, Comment

## Abstract

**Background:**

A recent meta-analysis by Posadzki et al. synthesized randomized controlled trials to evaluate the effectiveness and safety of spinal manipulative therapy (SMT) for migraines. Considering *Systematic Reviews* recognizes several methodological guidelines and reporting standards, our Letter highlights deviations from best practice methodologies.

**Main findings:**

We detail issues with the search strategy, application of selection criteria, inclusion of data, and outcome reporting and analysis. We partially replicated the authors’ search across three of their seven databases, which identified 1845 more articles than they reported. Finally, the authors’ interpretations appear to conflate mild and transient adverse effects with serious ones and minimize potentially meaningful benefits of SMT.

**Conclusion:**

The methodological limitations in the meta-analysis by Posadzki et al. raise concerns about its reliability and reproducibility. Accordingly, we advise against relying on this study to guide clinical decision-making. Clinicians, patients, and stakeholders should interpret its conclusions cautiously when evaluating the appropriateness of SMT for migraine management.

**Supplementary Information:**

The online version contains supplementary material available at 10.1186/s13643-025-02849-5.

## Main text

A recent meta-analysis by Posadzki et al. synthesized randomized controlled trials (RCTs) on spinal manipulative therapy (SMT) for migraine [1]. Considering *Systematic Reviews* recognizes several guidelines and reporting standards, we highlight methodological concerns in relation to best practices, including Cochrane, Preferred Reporting Items for Systematic reviews and Meta-Analyses (PRISMA), and Grading of Recommendations Assessment, Development, and Evaluation (GRADE) [2–6].

Issues with the search strategy and results raise concerns about validity and reproducibility. There was no comprehensive PRISMA flowchart with database-specific results, explanations/citations for excluded full-texts, indication of citation tracking, or method for gray literature [1]. With a research librarian, we partially replicated the search across three databases (i.e., Cochrane Central, Embase, and Ovid; Supplementary file 1). This retrieved 3307 articles (Figshare repository [7]), 1845 more than Posadzki et al. reported across seven databases, and we identified RCTs that were potentially missed or could have met their full-text eligibility (databases: [8, 9]; citation tracking: [10]).

The exclusion of RCTs using soft tissue techniques appears inconsistent. Rist et al. and Nelson et al. were included despite using “soft tissue release” [11], and “[5 to 10 min] of massage and/or trigger point therapy” [12], respectively. These discrepancies may bias the results, and prevent the effects of SMT from being isolated.

Relevant data were seemingly not included, thereby raising concerns about biased results. Demonstratively, Muñoz-Gómez et al. reported migraine frequency data using the Migraine Disability Assessment [13], yet their data were omitted without rationale.

Inconsistent outcome reporting complicates the statistical interpretation and raises questions about potentially biased results. Illustratively, without explaining conversions or assumptions, the authors reported a between-group change score for Rist et al. [11] but reported post-treatment means for other studies (e.g., Chaibi and Tuchin [14, 15]). Counterintuitively, Posadzki et al.’s forest plot approximated a null effect for Rist et al.’s migraine days outcome despite the original study reporting a mean change favoring SMT without overlapping the null [11].

The interpretation of adverse effects (AEs) is misleading due to lacking contextualization regarding AE severity. Although no included studies identified serious AEs [11–16], the authors overstated SMT’s risks by conflating mild AEs like tenderness with severe ones like “deaths.” For example, Chaibi et al. Reported more AEs in the SMT group versus placebo, yet transient local tenderness was the most common AE [14].The authors’ negative conclusions about SMT for migraine are not well supported. Despite wide confidence intervals, SMT was favored over control interventions across all treatment effects (i.e., migraine days, migraine duration, emotional quality of life, disability, and intensity/severity) [1]. The authors did not highlight reductions in migraine days and disability in the abstract or conclusions, despite these favoring SMT without crossing the null. Furthermore, claiming that “effectiveness of SMT for the treatment of migraines remains unproven” misrepresents the role of systematic reviews, which provide evidence for or against a conclusion rather than proof [5, 17].

Given these concerns, patients, clinicians, and stakeholders should be cautious when interpreting the study and determining whether SMT is effective or safe for migraines.

## Supplementary Information

Supplementary Material 1. Three databases.

## Data Availability

The Research Information Systems files and deduplicated spreadsheet of articles that support our partial search replication are available in Figshare (https://doi.org/10.6084/m9.figshare.28057199 [7]).

## Abbreviations

AE: Adverse effects
GRADE: Grading of Recommendations Assessment, Development, and Evaluation
RCT: Randomized controlled trial
SMT: Spinal manipulative therapy
