# Supplementary material for: Critical limitations compromise the conclusions of a recent meta-analysis regarding spinal manipulation and migraine: a commentary
Source: Syst Rev. 2025 Oct 23;14:200. doi: 10.1186/s13643-025-02849-5 (PMC12548160; doi:10.1186/s13643-025-02849-5)
Supplement: Supplementary file 1 — Supplementary Material 1. Three databases. [file 13643_2025_2849_MOESM1_ESM.pdf]

Cochrane 12/16/2024

Search Name: Trager-Headache and Chiropractic Medicine

Date Run: 16/12/2024 12:54:11

Comment: #26, #27, #28-searched all text to match ".mp" from original search

ID	Search	Hits
#1	MeSH descriptor: [Migraine Disorders] this term only	3794
#2	(Migraine*):ti OR (Migraine*):ab	9738
#3	(vascular headache):ti OR (vascular headache):ab	514
#4	(hemicrania headache):ti OR (hemicrania headache):ab	16
#5	(cephalalgia headache):ti OR (cephalalgia headache):ab	28
#6	MeSH descriptor: [Headache Disorders, Primary] this term only	31
#7	MeSH descriptor: [Headache] this term only	3237
#8	#6 OR #7	3250
#9	#1 OR #2 OR #3 OR #4 OR #5 OR #8	12743
#10	MeSH descriptor: [Manipulation, Spinal] this term only	559
#11	MeSH descriptor: [Chiropractic] this term only	205
#12	MeSH descriptor: [Manipulation, Chiropractic] this term only	185
#13	("spinal manipulation"):ti OR ("spinal manipulation"):ab	758
#14	("spinal adjustment"):ti OR ("spinal adjustment"):ab	13
#15	("cervical manipulation"):ti OR ("cervical manipulation"):ab	129
#16	("cervical adjustment"):ti OR ("cervical adjustment"):ab	4
#17	("chiropractic manipulation"):ti OR ("chiropractic manipulation"):ab	101
#18	("chiropractic adjustment"):ti OR ("chiropractic adjustment"):ab	30
#19	MeSH descriptor: [Manipulation, Osteopathic] this term only	224
#20	MeSH descriptor: [Osteopathic Medicine] explode all trees	42
#21	MeSH descriptor: [Musculoskeletal Manipulations] explode all trees	4466
#22	MeSH descriptor: [Complementary Therapies] explode all trees	30095

#23 MeSH descriptor: [Complementary Therapies] explode all trees 30095

#24 (manual NEXT therap*):ti OR (manual NEXT therap*):ab 2614

#25 (Osteopath*):ti OR (Osteopath*):ab 1092

#26 (osteopath* manipulation*) OR (osteopath* manipulation*) 590

#27 (osteopath* NEXT therap*) OR (osteopath* NEXT therap*) 20

#28 (osteopath* NEXT treatment*) OR (osteopath* NEXT treatment*) 250

#29 (High velocity thrust*):ti OR (High velocity thrust*):ab 160

#30 (Subluxation*):ti OR (Subluxation*):ab 592

#31 #10 OR #11 OR #12 OR #13 OR #14 OR #15 OR #16 OR #17 OR #18 OR #19 OR #20 OR #21 OR #22 OR #23 OR #24 OR #25 OR #26 OR #27 OR #28 OR #29 OR #30 35058

#32 #9 AND #31 597

#33 limit 32 to yr “2010-2023”, English only: 279 trials

limit 32 to yr “2010-2023”, English only: 7 reviews

OVID 12/16/2024

Ovid MEDLINE(R) ALL <1946 to December 13, 2024>

1 Migraine Disorders/ 31256

2 Migraine$.ti,ab.44464

3 (headache* adj3 (vascular or hemicrania or cephalalgia)).ti,ab. 985

4 Headache Disorders, Primary/ or Headache/ 33632

5 1 or 2 or 3 or 4 76378

6 Manipulation, Spinal/ 1894

7 Chiropractic/ 3547

8 Manipulation, Chiropractic/ 1145

9 ((spinal or cervical or chiropractic) adj3 (manipulat$ or adjust$)).ti,ab. 4119

10 Manipulation, Osteopathic/ 1273

11 exp Osteopathic Medicine/ 3439

12 exp Musculoskeletal Manipulations/ 19444

13	exp alternative medicine/	256062
14	exp Complementary Therapies/	256062
15	(manual adj2 therap$).ti,ab.	4074
16	Osteopath$.ti,ab.	6928
17	(Osteopath$ adj3 manipulat$).mp.	1877
18	(Osteopath$ adj5 therap$).mp.	539
19	(Osteopath$ adj5 treatment).mp.	1074
20	High velocity thrust$.ti,ab.	29
21	Subluxation$.ti,ab.	11792
22	6 or 7 or 8 or 9 or 10 or 11 or 12 or 13 or 14 or 15 or 16 or 17 or 18 or 19 or 20 or 21	286488
23	5 and 22	2566
24	limit 23 to (english language and yr="2010 - 2023")	800

EMBASE 12/16/2024

Embase

Session Results

…………………………………………………

No.	Query Results	Results	Date
#20.	#18 AND (2010:py OR 2011:py OR 2012:py OR 2013:py OR 2014:py OR 2015:py OR 2016:py OR 2017:py OR 2018:py OR 2019:py OR 2020:py OR 2021:py OR 2022:py OR 2023:py) AND [english]/lim	2,228	16 Dec 2024
#19.	#18 AND (2010:py OR 2011:py OR 2012:py OR 2013:py OR 2014:py OR 2015:py OR 2016:py OR 2017:py OR 2018:py OR 2019:py OR 2020:py OR 2021:py OR 2022:py OR 2023:py)	2,298	16 Dec 2024
#18.	#1 AND #17	3,624	16 Dec 2024
#17.	#2 OR #3 OR #4 OR #5 OR #6 OR #7 OR #8 OR #9 OR #10 OR #11 OR #12 OR #13 OR #14 OR #15 OR #16	168,916	16 Dec 2024

#16. 'subluxation*':ab,ti	14,118	16 Dec 2024
#15. 'high velocity thrust*':ab,ti	41	16 Dec 2024
#14. 'osteopath* treatment*':ab,ti,kw	429	16 Dec 2024
#13. 'osteopath* therap*':ab,ti,kw	58	16 Dec 2024
#12. 'osteopath* manipulation*':ab,ti,kw	293	16 Dec 2024
#11. 'osteopath*':ti,ab	9,401	16 Dec 2024
#10. 'manual therap*':ab,ti	4,672	16 Dec 2024
#9. 'alternative medicine'/exp	82,953	16 Dec 2024
#8. 'musculoskeletal manipulation'/exp	5,626	16 Dec 2024
#7. 'osteopathic medicine'/exp	6,180	16 Dec 2024
#6. 'osteopathic manipulation'/exp	807	16 Dec 2024
#5. 'spinal manipulation':ab,ti OR 'spinal adjustment':ab,ti OR 'cervical manipulation':ab,ti OR 'cervical adjustment':ab,ti OR 'chiropractic manipulation':ab,ti OR 'chiropractic adjustment':ab,ti	2,534	16 Dec 2024
#4. 'chiropractic manipulation'/exp	445	16 Dec 2024
#3. 'chiropractic'/exp	5,557	16 Dec 2024
#2. 'spine manipulation'/exp	1,236	16 Dec 2024
#1. 'migraine'/exp OR 'primary headache'/exp OR 'headache'/exp OR 'migraine*':ab,ti OR 'vascular headache*':ab,ti OR 'hemicrania headache*':ab,ti OR 'cephalalgia headache*':ab,ti	368,985	16 Dec 2024

……………………………………………….
